# Characterization of endogenous antioxidant attributes and its influence on thermal stability of canola oil†

**DOI:** 10.1039/c8ra02275e

**Published:** 2018-10-23

**Authors:** Wenting Shang, Huijuan Dong, Padraig Strappe, Zhongkai Zhou, Chris Blanchard

**Affiliations:** Key Laboratory of Food Nutrition and Safety, Ministry of Education, School of Food Engineering and Biotechnology, Tianjin University of Science and Technology Tianjin 300457 China zkzhou@tust.edu.cn +862260601371 +862260601408; School of Medical and Applied Sciences, Central Queensland University Rockhampton Qld 4700 Australia; ARC Industrial Transformation Training Centre for Functional Grains, Charles Sturt University Wagga Wagga NSW 2678 Australia

## Abstract

Difference in thermal stability of two commercially available canola oils prepared by either expeller-extraction (EE) or solvent-extraction (SE) method was investigated. After 5 days consecutive deep-fry, content of oxidized-triacylglycerols (oxTAGs) in SE oil increased by 250.0% compared to its original status. However, 62.5% increase of oxTAGs in EE oil occurred, indicating that EE oil exhibits superior thermal stability to SE oil. Antioxidant capacity of EE oil was highly retained and loss rate of tocopherols in EE oil was much slower than in SE oil during deep-fry. Lipidomics showed that although there was no significant difference in molecular profile of either triacylglycerols or diacylglycerols between two oils, EE oil was characterized with 19 times higher phosphatidylcholine contents than SE oil. Considering no difference in antioxidant capacity between the two oils in their original status, it is proposed that synergetic mechanism is simultaneously initiated by antioxidant compounds and phosphatidylcholines, which plays key roles for maintaining better thermo-stability of vegetable oil during deep-fry.

## Abstract 2

Difference in thermal stability of two commercially available canola oils prepared by either expeller-extraction (EE) or solvent-extraction (SE) method was investigated.

## Graphical abstract

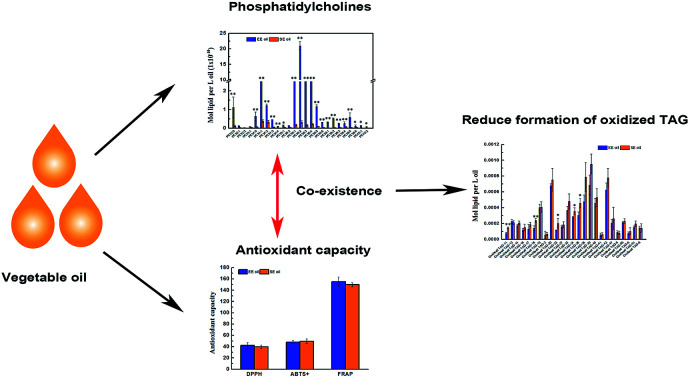

## 1 Introduction

Canola oil is one of the most common vegetable oils used in dietary supplementation. The composition of oil constituents, such as tocopherols, phytosterol and phospholipids, is influenced by the oil extraction process,^1^ which can also affect oil physicochemical properties. Deep-fry is one of the most popular cooking methods, and thermal-oxidation occurs during the deep-frying process and is an important phenomenon associated with oil deterioration. Thermal-oxidation results in the formation of volatile and nonvolatile decomposition compounds which inevitably affect the quality of the frying oil.^2–4^ Furthermore, the repeated use of oil at high temperature reduces the shelf life of frying oil and may impact human health.^5^

Deep-fry leads to an increased level of total polar compounds (TPC), and these components are measured as non-volatile and represent one of the major reactions occurred during the frying process.^6^ Research has shown that the polar compounds isolated from fried oil induced cytotoxicity in HepG2 cells.^7^ Thus, the measurement of TPC is an important parameter for evaluation of the changes in oil quality during the frying process.^8,9^

Polar compounds as complex mixtures primarily involve interaction between oxygen and oil molecules. These compounds consist of oxidised triacylglycerols (oxTAGs) formed through the oxidative alteration of triglycerides. Free fatty acids (FFA) and diacylglycerols (DAGs) are formed through hydrolytic process of triglycerides, while dimeric triacylglycerols and polymeric triacylglycerols are formed through thermal polymerization of triglycerides.^10^ Yoon and Jung (1988)^11^ reported that the oxTAGs and some oxidized compounds which act as pro-oxidants can influence the oxidative stability of the oil, highlighting the importance of oxTAGs as important indicators for oil quality.

Other studies have also shown that frying of oil is associated with the properties that show resistance to oxidation performance, which are dependent on fatty acid composition, in particular the degree of unsaturation, the distribution of triacylglycerols,^12^ and the concentration of bioactive compounds such as phenolics, sterols and tocopherols.^13–15^ Canola oil is rich in monounsaturated and polyunsaturated fatty acids (MUFA and PUFA) with a low content of saturated fatty acids (SFA).^16^ Fatty acid composition alone does not completely explain the thermal stability of fried oils. Other compounds, in particular some minor components such as tocopherols and phospholipids were reported to be favorable to thermal-oxidation stability of oil during frying.^17,18^ However, the factors influencing the thermal-oxidation stability of canola oil have not been fully elucidated. Particularly, the study of the effect of different extraction technologies on oil thermal-oxidation stability is rare. Therefore, the aim of this study is to compare the thermal properties of two canola oils produced through either solvent-extraction or a physically based oil extraction method known as expeller-extraction. The two oils are evaluated following the deep-frying model and the factors affecting the oil thermal stability are analyzed.

## 2 Materials and methods

### 2.1 Materials

Canola oil was extracted and purified from the same Australian variety (*Brassica napus*) using two extraction technologies, respectively: expeller-extraction (a physical extraction method, referred to as EE oil) and solvent-extraction (a traditional oil extraction method, referred as SE oil). Both products are commercially available in Australian supermarket and are being used as vegetable oils. Reference chemicals such as α, β, γ, and δ-tocopherols were purchased from Sigma-Aldrich Supelco Co. (USA). Potatoes were bought from a local supermarket (Tianjin, China). All solvents and chemicals used in this study were of analytical and HPLC grades.

### 2.2 Deep-frying procedure

Fresh potatoes were peeled and sliced to certain specification chips using a potato chipper (Baimao, Zhejiang, China) and were stored at −80 °C. The frying process was carried out in a 5 liter stainless steel deep fryer (Winterrain, Australia) containing 4 L oil in the container heated to 185 ± 5 °C. A batch of frozen chips (100 g on the first day which was reduced by 10 g each in the following day) was poured into the heated oil for 1.5 min each time until golden color. After 30 min, this process was repeated. A total of 9 batches of potatoes were fried using above condition each day for 5 consecutive days. At the end of each day, oil samples were allowed to cool to room temperature, and then 50 mL samples were collected for analysis and the oil was not replenished throughout the whole frying process.

### 2.3 Analysis of total polar compounds (TPC)

The total amount of polar compounds was analyzed using a published method with minor modifications.^19^ Briefly, oil sample (1.0 g) was dissolved in petroleum ether (5 mL). Then the mixture was analyzed on an Edible Oil Polar Components Rapid Preparation of Column Chromatography System (EOPC PFC SYSTEM) equipped with an EOPC FLASH Chromatographic Column (Bonna-Agela Technology Company, Tianjin, China) and UV-detector. The mobile phase was the eluent of non-polar components with petroleum ether and diethyl ether (87 : 13, v/v). Detection was carried out at 200 nm and a flow rate of 25 mL min^−1^. The solvent in the non-polar compounds fraction was evaporated using a rotary evaporator at 60 °C and then the residue was kept in the vacuum oven at 40 °C for 20–30 min for further drying prior to analysis. Finally, the percentage of TPC was calculated based on the weight of the remaining non-polar compounds.

### 2.4 Fatty acids composition analysis

The two canola oils were characterized as fatty acid methyl esters following derivatization with 14% (w/v) boron trifluoride in methanol.^20^ The fatty acid composition of oil samples was determined by gas chromatography-mass spectrometry (GC-MS) and was expressed in relative area percentages. Fatty acid methyl esters (FAMEs) were prepared and analyzed on a Varian 4000 GC-MS using a VF-5 ms (30 m × 0.25 mm × 0.25 μm) capillary column. The carrier gas was set at 1 mL min^−1^ flow rate, and the column temperature was programmed from 70 °C to 100 °C at a rate of 5 °C min^−1^ and held for 2 min, then was increased to 175 °C at 10 °C min^−1^ and held for 40 min, followed by the rise to 220 °C at a rate of 15 °C min^−1^. The initial and final temperatures were held for 1 and 30 min, respectively. The temperature of injector and detector was set at 260 °C.

### 2.5 Tocopherol analysis

The content of tocopherols in the oil was analyzed using a method described previously.^21^ Briefly, oil samples (0.5 g) were dissolved in 5 mL *n*-hexane, and the mixture was sonicated for 15 min and centrifuged at 10 000 rpm for 10 min. Samples were filtered through a 2.5 μm membrane and the tocopherols were detected using a high performance liquid chromatography system (Shimadzu, Tokyo, Japan) equipped with a Waters XTerra® RP 18 column (250 mm × 4.6 mm, 5 μm particle size) with column temperature maintained at 40 °C. The mobile phase was methanol/water (94 : 6) with isocratic elution at a flow rate of 1 mL min^−1^. The DAD-detection was performed at 295 nm.

### 2.6 Determination of phytosterols

Saponified oil samples were prepared according to the published method.^22^ In brief, 0.03 g oil sample was mixed with 3 mL of 2 mol L^−1^ KOH in 95% ethanol in a glass tube and shaken in a water bath at 90 °C for 15 min. The internal standard (150 μL of 0.5 mg mL^−1^ β-cholestanol in *n*-hexane) was added to the sample before saponification. After cooling, 2 mL of water and 1.5 mL of hexane were added and mixed vigorously. The mixture was then centrifuged at 5000 rpm for 15 min, and the hexane layer containing unsaponifiables was separated for further analysis. A GC (Varian 4000 GC/MS) with a VF-5 ms column (30 m × 0.25 mm id, 0.25 μm film thickness) was used for the analysis. The oven temperature was programmed from 150 °C (held for 3 min) to 300 °C at 5 °C min^−1^ and held for 10 min. Identification of the individual phytosterols was accomplished by NIST05 mass spectral library.

### 2.7 Lipidomic analysis of molecular profile in canola oils

Glycerol lipids (phosphatidylcholines, triacylglycerides and diacylglycerides) were analyzed using a modified version of reverse phase (RP)-HPLC/ESI/MS/MS described previously^23^ on a Shimadzu Exion UPLC coupled with SCIEX 6500 QTRAP Plus system. Briefly, samples for neutral lipid analysis were prepared by dissolving oil with chloroform : methanol (1 : 1) solvent. The phosphatidylcholines (PCs) were concentrated for analysis by extraction with analytical grade ethanol. Separation of the aforementioned lipids was carried out on a Phenomenex Kinetex 2.6μ-C18 column (internal diameter 4.6 × 100 mm) using an isocratic mobile phase chloroform : methanol : 0.1 mol L^−1^ ammonium acetate (100 : 100 : 4) at a flow rate of 160 μL min^−1^ for 20 min. Using neutral loss-based MS/MS techniques, the levels of TAGs were calculated as relative contents to the spiked d5-TAG 48:0 internal standard (CDN Isotopes), while DAG species were quantified using 4ME 16:0 diether DG as an internal standard (Avanti Polar Lipids, Alabaster, AL, USA).

### 2.8 Sample preparation of antioxidant capacity

The canola oil extracts were formulated in methanol according to a previously described procedure.^24^ The test tubes with oil sample (3.00 g) and methanol (5 mL) were shaken for 30 min at room temperature in the dark. Then the extracts were separated from oils following freezing (−20 °C for 30 min) and transferred quantitatively into a glass bottle. Each oil sample was extracted in triplicate and the extracts were stored in a refrigerator prior to antioxidant capacity analysis.

### 2.9 DPPH scavenging activity

The DPPH assay was performed to estimate the scavenging activity of above extracts according to a method adapted from Szydłowska-Czerniak *et al.* (2010).^25^ In brief, 0.2 mL of oil extract was added to 1.3 mL of methanol and 0.5 mL of DPPH˙ methanolic solution (304.0 μmol L^−1^). The mixture was shaken vigorously and left to stand at room temperature for 30 min in darkness. The absorbance was measured at 517 nm against a reagent blank (1.5 mL of methanol + 0.5 mL of DPPH methanolic solution). The DPPH scavenging activity was calculated as follows:
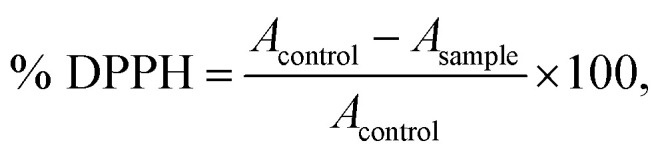
where *A*_control_ = absorbance of DPPH radical + methanol; *A*_sample_ = absorbance of DPPH radical + oil extracts.

### 2.10 ABTS inhibition activity

The ABTS free radical-scavenging activity of each sample was determined.^26^ The ABTS˙^+^ solution (7 mmol L^−1^ ABTS stock solution with 2.45 mmol L^−1^ potassium persulfate 1 : 0.5 was kept for 12–16 h before use in the dark at room temperature) was diluted with ethanol to an absorbance of 0.70 (±0.02) at 734 nm. The oil extract of 0.3 mL was added to 2.2 mL of diluted ABTS˙^+^ solution and the mixtures were incubated at 30 °C for 10 min. The absorbance was measured at 734 nm against a reagent blank (2.5 mL of ABTS˙^+^ solution). The percentage of ABTS inhibition was calculated as follows:
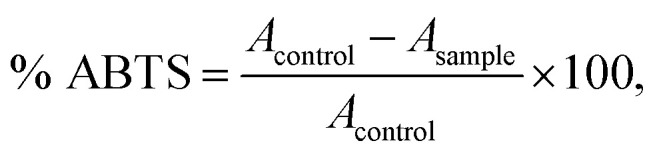
where *A*_control_ = absorbance of ABTS radical + methanol; *A*_sample_ = absorbance of ABTS radical + oil extracts.

### 2.11 FRAP capacity

The antioxidant capacity of the oil samples was conducted by the FRAP method with some modifications.^27^ The freshly prepared FRAP reagent (2.5 mL of 10 mmol L^−1^ TPTZ solution in 40 mmol L^−1^ HCl, 2.5 mL of 20 mmol L^−1^ FeCl_3_ and 25 mL of 0.1 mol L^−1^ acetate buffer, pH 3.6) was incubated at 37 °C for 10 min in a water bath. Then, 20 μL of methanolic extract of the oil samples and 400 μL of FRAP reagent mixture were mixed and kept at 37 °C for 4 min. The solution was then centrifuged at 10 000 rpm for 5 min to remove solids. The absorbance was measured at 593 nm against a reagent blank (400 μL of FRAP reagent and 20 μL methanol).

### 2.12 Statistical analysis

The obtained results are presented as mean ± standard deviation (SD). Data were analyzed by *T*-test analysis using SPSS statistics 19.0. The differences were considered statistically significant at the 95% level (*P* < 0.05).

## 3 Results and discussion

### 3.1 Lipids molecular characterization of the oils

**Fig. 1:**
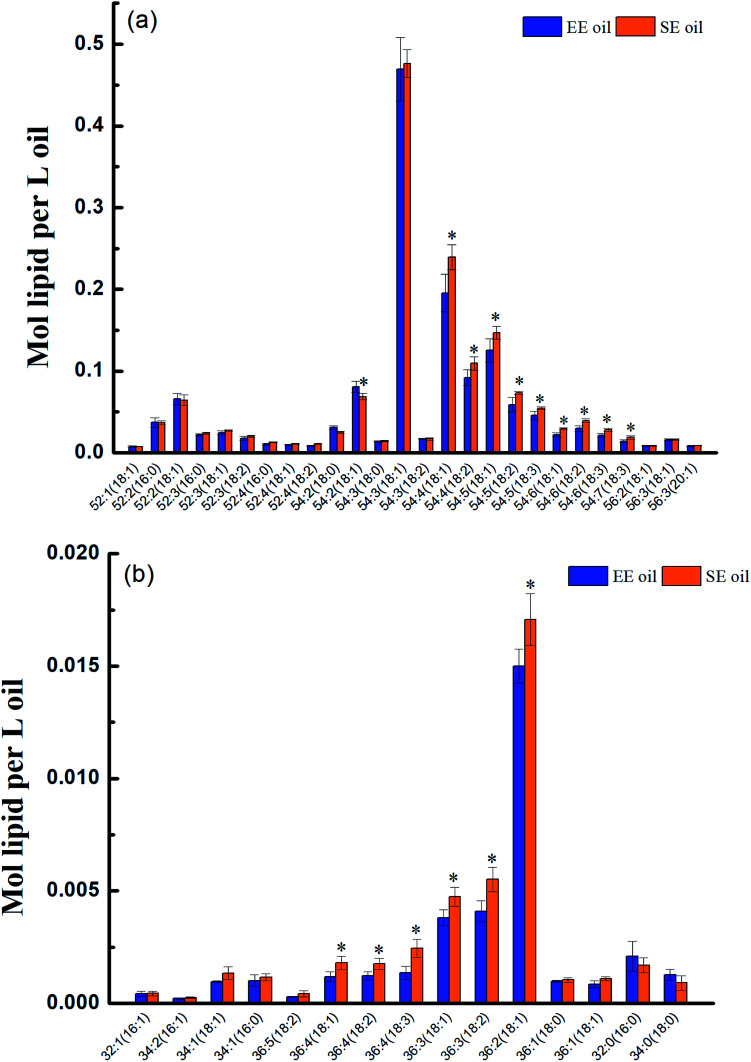
Molecular characteristics of TAGs and DAGs in the two oils. (a) TAGs; (b) DAGs. “*” *P* < 0.05 *versus* control (SE oil).

The molecular profile of two major constituents, TAG and DAG in the oil, was measured by lipidomics and the data are presented in Fig. 1. Two hundred fifty nine species of TAG were determined in both oils, and they were characterized with the dominant species of 54 : 3 (18 : 1), 54 : 4 (18 : 1), 54 : 5 (18 : 1), 54 : 4 (18 : 2), 54 : 5 (18 : 2) and 54 : 5 (18 : 3) in the TAG group. Nevertheless, some difference in the TAG profile between the two oils was still noted, in which SE oil demonstrated higher content in 54 : 4 (18 : 1), 54 : 5 (18 : 1), 54 : 4 (18 : 2) compared to EE oil. In contrast, species number of DAG is much less than that of TAG, in which just 15 species were determined in the two oils (Fig. 1b). It was interesting to see that the difference in the DAG profile between the two oils focused on unsaturated fatty acids, such as 36 : 2 (18 : 1), 36 : 3 (18 : 2), 36 : 4 (18 : 3), and not on saturated fatty acids.

### 3.2 Effect of deep-fry on lipids molecular characterization of the oils

**Fig. 2:**
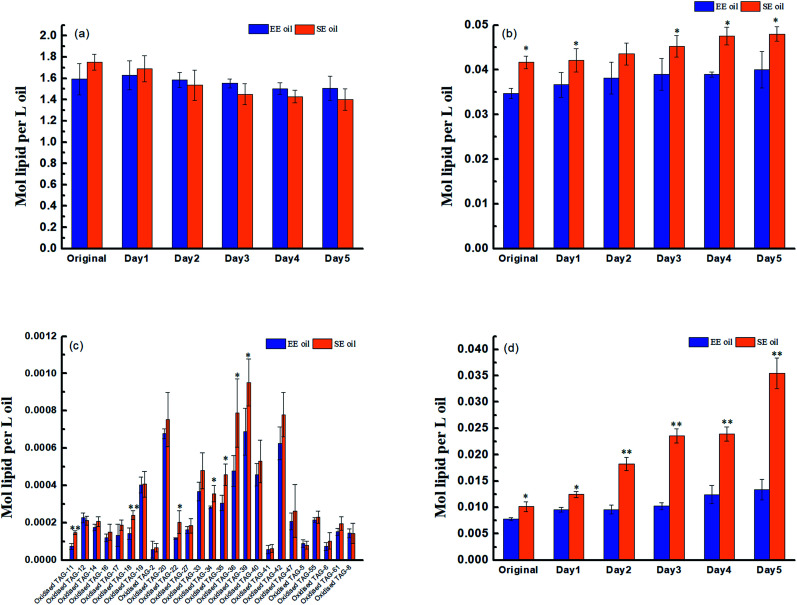
Effect of deep-fry on lipids molecular characterization. (a) TAGs; (b) DAGs; (c) molecular profile of oxidized TAGs; (d) total content of oxidized TAGs. “*” *P* < 0.05 *versus* control, and “**” *P* < 0.01 *versus* control.

Prior to deep-fry, the molecular profile of either TAGs or DAGs between the two oils demonstrated a higher similarity (Fig. 2a and b). The data also showed that deep-fry led to a slight reduction of TAG content and an increase in DAG content in the two oils, which may indicate a conversion of TAGs to DAGs during the deep-fry. Furthermore, the measurement of the oxidative products of TAGs (oxTAGs) in the oils indicated that 62 species were noted in the current study (Fig. 2c). Prior to deep-fry, the total level of oxTAGs was very close between the two oils (0.008 *versus* 0.010 mol L^−1^). More importantly, this study found that deep-fry led to a rapid increase in oxTAG content for SE oil, but the changes in total oxTAGs content in the EE oil was found to be steady during the deep-frying process (Fig. 2d). After the 5 days deep-fry, the total content of oxTAGs in SE oil was promoted to 0.035 mol L^−1^ with a 250.0% increase compared to its original status (before deep-fry). However, only 62.5% increase of oxTAGs in EE oil after the deep-frying procedure was found in this study. This difference in the formation of thermo-oxidative products caused by deep-frying process between the two oils may be associated with their corresponding molecular characteristics.

### 3.3 Effect of deep-fry on total polar components

**Fig. 3:**
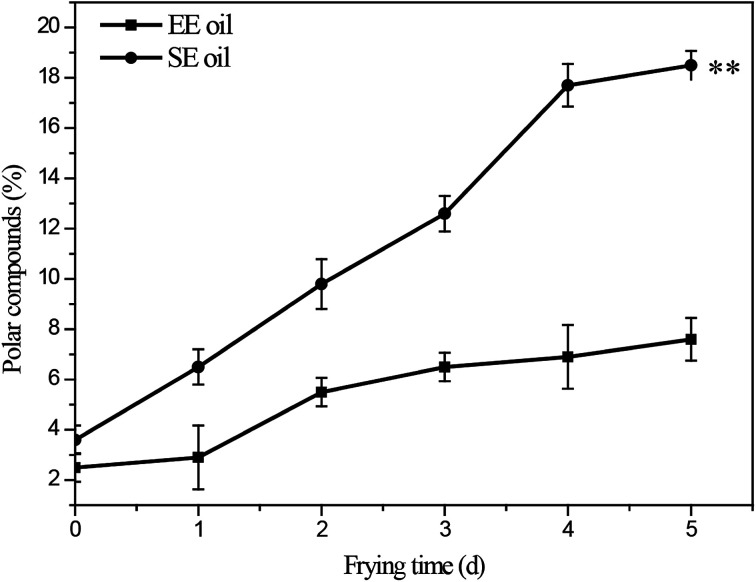
Changes in total content of polar compounds during deep-frying process. “**” *P* < 0.01 *versus* control at the end of 5 days deep-fry.

The influence of deep-frying process on the changes in the content of total polar components (TPC) is presented in Fig. 3. The fresh canola oils from two different extraction methods had a TPC content of 2.5% (EE oil) and 3.6% (SE oil), respectively. In contrast, at the end of the frying period, TPC content increased to 7.6% and 18.5% for EE and SE oils, respectively (Fig. 3). This is consistent with a previous study, in which a repeated frying led to an increased TPC content of sunflower seed oil.^2^ More importantly, this study found that the rate of the increase in TPC content following each frying time was significantly (*P* < 0.05) higher in SE oil compared to EE oil (Fig. 3), suggesting that EE oil exhibits far superior heat stability to SE oil.

### 3.4 Fatty acid composition analysis

In order to investigate the factors contributing to the different thermal stability between the two oils during deep-frying process, the fatty acid composition was analyzed and the results are presented in Table S1,† with oleic (C18:1), linoleic (C18:2), palmitic (C16:0) and stearic (C18:0) acid as the most predominant fatty acids in both oils. The two oils in their corresponding non-fried status showed some differences in the proportion of SFA (saturated fatty acid), MUFA (monounsaturated fatty acid) and PUFA (polyunsaturated fatty acid), in which EE oil had higher level of PUFA, but lower level of MUFA compared to SE oil. A previous study suggested that the oils with a higher ratio of UFA are prone to oxidation.^19^ However, our results demonstrated that the EE oil has a higher thermal-oxidation stability compared to SE oil, suggesting that other constituents in the oil, in particular some minor compounds (*e.g.* antioxidant compounds, *etc.*) may provide the oil molecular stability during the frying process.

### 3.5 Analysis of antioxidant capacity of canola oils

Considering that canola oil contains beneficial bioactive compounds such as polyphenols, sterols, tocopherols and flavonoids, which play roles in protection against oxidation of the oil molecules,^28–30^ thus, their corresponding antioxidant capacity was measured and the data are presented in Table 1. This study revealed that the oil extraction methods used in this study did not result in significant differences (*P* > 0.05) in antioxidant capacity between the two oils (non-fried status) as analyzed by DPPH, ABTS^+^ and FRAP assays, although the antioxidant capacity of EE oil was slightly higher than that of SE oil. However, the results also showed that after 1 day of frying, the antioxidant capacity of the SE oil was significantly decreased compared to EE (*P* < 0.05). More importantly, this study found that the antioxidant capacity of EE oil was still highly retained following the deep-frying process. In contrast, SE oil lost its antioxidant capacity quickly during the process. The data from Table 1 showed that the DPPH, ABTS^+^ and FRAP results for SE oil had an 88.1%, 62.6%, and 78.7% reduction, respectively, compared to a corresponding reduction of 32.5%, 14.4%, and 24.4% in EE oil at the end of 5 days deep-frying, indicating that the antioxidant compounds in the two oils demonstrate different resistance to the thermal treatment. Thus, the analysis of the difference in the content and profile of the key antioxidant compounds between the two oils becomes essential.

**Table 1:** Changes in antioxidant capacity of two oils during deep-frying process^a^

Deep-fry time (d)	Antioxidant capacity		
	DPPH	ABTS˙^+^	FRAP
**EE oil**			
0	42.19 ± 5.16^a^	48.09 ± 2.89^a^	155.08 ± 8.23^a^
1	39.38 ± 1.36^ab^	49.25 ± 4.80^a^	136.99 ± 6.48^b^
2	35.19 ± 0.63^bc^	47.31 ± 3.19^a^	137.47 ± 2.18^b^
3	34.10 ± 2.96^c^	45.23 ± 5.46^a^	141.035 ± 7.86^b^
4	30.33 ± 1.29^cd^	45.24 ± 4.39^a^	139.61 ± 12.04^b^
5	28.47 ± 1.70^d^	41.15 ± 3.88^a^	117.24 ± 5.15^c^

**SE oil**			
0	39.67 ± 2.99^a^	49.46 ± 4.14^a^	149.60 ± 3.53^a^
1	23.83 ± 4.68^b^	30.04 ± 4.16^b^	96.05 ± 8.93^b^
2	20.17 ± 3.14^b^	24.10 ± 0.511^c^	63.93 ± 8.58^c^
3	12.23 ± 0.91^c^	19.73 ± 0.28^cd^	44.89 ± 5.41^d^
4	7.16 ± 2.42^d^	19.91 ± 1.67^cd^	38.94 ± 2.70^d^
5	4.74 ± 1.02^d^	18.50 ± 0.94^d^	31.80 ± 1.49^d^

a Values are expressed as mean ± standard deviations (*n* = 3). Different letters are significant in the same column at *P* < 0.05.

### 3.6 Tocopherols content in canola oils

**Fig. 4:**
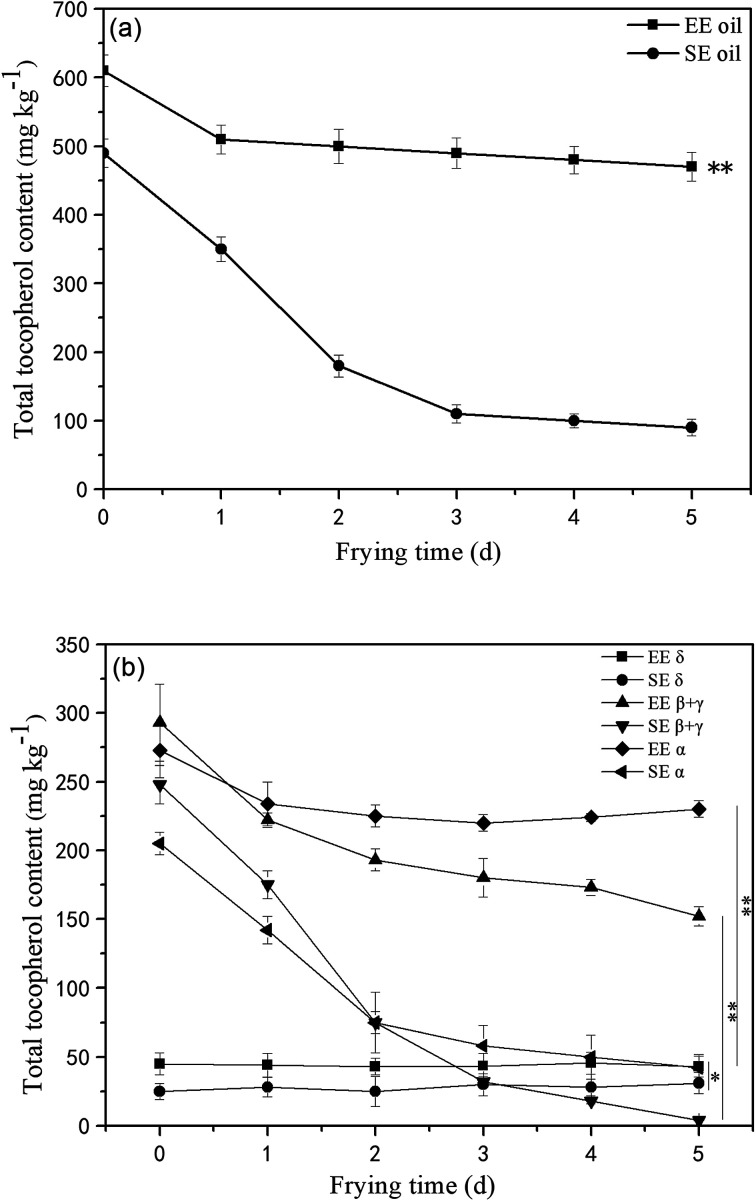
Loss of total and individual tocopherols in the oils during deep-fry (calculated by individual δ-tocopherol, β-tocopherol, γ-tocopherol and α-tocopherol from HPLC measurement). (a) Total tocopherols; (b) individual tocopherols. “**” *P* < 0.01 *versus* control at the end of 5 days deep-fry.

Tocopherols, as members of the vitamin E family, are important constituents in the oils and are recognized as potent lipophilic antioxidants, which can be used as an indication of frying oil stability by slowing down the oxidative degradation progress of the oil molecules.^18^ This study found that the total amount of initial tocopherols content was significantly higher (*P* < 0.05) in EE oil than that in SE oil, indicating the influence of the oil extraction process on the tocopherol content in the final oil sample (Fig. 4a). The study on the effect of deep-frying process on the total tocopherols content revealed that the loss of tocopherols was clearly at a much slower rate in EE oil compared to SE oil during the 5 days frying, which may suggest that tocopherols in EE oil provides a stronger protective effect on oil stability during the deep-frying, and the pattern of the changes in the total tocopherols content following deep-frying process seems to be highly consistent with their corresponding thermo-oxidative stabilities of the two oils as shown in Fig. 3. Rossi *et al.*^31^ also reported that tocopherol is an excellent free radical scavenger, which can be used as a hydrogen donor to remove the original free radical, and at the same time generate a relatively stable free radical intermediate which may be associated with the oils exhibiting a greater anti-oxidative ability.

Furthermore, the tocopherols profile was also analyzed (Fig. 4b) showing that the proportion of individual tocopherol follows the order: β + γ- > α- > δ- in the two canola oils. This is the first report to investigate the effect of deep-frying on the loss of individual tocopherol in canola oil, in which δ-tocopherol was found to have a better stability than others during heating. In contrast, the content of β + γ and α-tocopherols reduced much faster, particularly in SE oil. Previous study suggested that tocopherols deteriorate gradually with extended frying time, and this phenomenon could negatively influence the protection roles on frying oil stability.^32^ Furthermore, this study found that the loss rate of tocopherols in EE oil is much lower than that in SE oil, which may suggest that other compounds in the EE oil provide much greater protection for the stabilization of tocopherols in EE oil whereas this occurred less in SE oil during the deep-fry.

### 3.7 Phytosterols content in canola oils

To understand the different stabilization property of tocopherols between the two oils during the fry, the content of individual phytosterol (brassicasterol, campesterol and β-sitosterol) in the two oils was analyzed and the results are presented at Fig. S1.† Phytosterols were reported to contribute to anti-oxidative function and thermal stability in vegetable oils^33^ and the current results indicated that β-sitosterol was the predominant phytosterol, followed by campesterol and brassicasterol. The EE oil had a higher content of β-sitosterol compared with SE oil, but no significant difference in the content of campesterol and brassicasterol between the oils, demonstrating the extraction efficiency of the expeller method for phytosterols, in particular for β-sitosterol.

### 3.8 Phosphatidylcholines content in canola oil

**Fig. 5:**
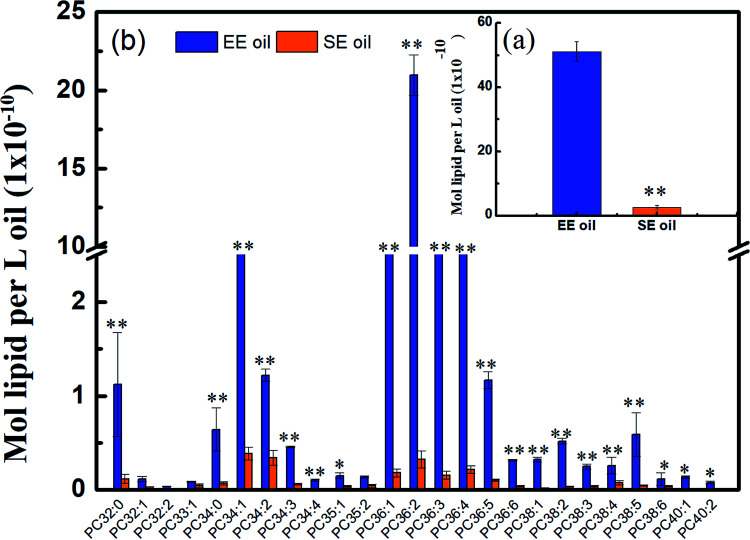
Different content of phosphatidylcholines in two canola oils. (a) Total content of PCs; (b) distribution of PCs. PC: phosphatidylcholine. “*” *P* < 0.05 *versus* control and “**” *P* < 0.01 *versus* control.

Considering that phospholipids (PCs) in the vegetable oil were reported to act as antioxidant synergists, which may enhance or extend the effect of tocopherol radical scavenging activity and enrich the anti-oxidant activity,^34,35^ the level of phosphatidylcholines (PCs) in the two oils is displayed in Fig. 5. Lipidomic analysis indicated that 43 species were found in their original oils, and the PCs with medium molecular size such as PC36:4, PC36:3, PC36:2, PC36:1 and PC34:1 are the dominant PC constituents, demonstrating much higher levels in EE oil than in SE oil (*P* < 0.05). More importantly, the PCs content in EE oil was 19 times higher than that in SE oil, indicating the impact of oil extraction procedure on oil constituents.

This outstanding difference in the PCs content and profile between the two oils may be due to the low solubility of the phospholipids during the solvent-extraction, leading to a much lower content in the final oil product (*i.e.* SE oil). Considering that the loss rate of tocopherols in EE oil during deep-frying is much lower than that in SE oil, the plausible interpretation is that the phosphatidylcholines may function as an antioxidant synergist^17^ for enhancing the thermo-oxidative stability of tocopherols compounds in EE oil during deep-frying, which occurred less in SE oil due to its lower content of PCs. Previous studies also indicated the existence of synergism between tocopherols and phospholipids for suppressing lipid oxidation. For example, lecithin with high proportions of phosphatidylcholines (PCs) and phosphatidylethanolamines (PEs) increased the activity of γ- and δ-tocopherols,^36^ and the combination of PCs, PEs and cardiolipin enhanced the activity of α-tocopherol.^37^ One possible mechanism for interpreting the synergistic activity of phospholipids and tocopherols may be due to the function of PCs and PEs, which are characterized with headgroups for regenerating tocopheroxyl radical to tocopherol.^38^ At the end, the effect of synergistic characteristics between tocopherols and phospholipids plays one of the key roles for benefiting the thermo-property of EE oil with a delayed/reduced formation of polar components and oxTAGs during the deep-frying.

## 4 Conclusions

This study revealed that canola oil extracted by the expeller method showed greater thermal-oxidation stability than oil extracted using a common solvent based method in terms of reduced levels of TPC and oxTAGs during deep-fry. More importantly, the loss rate of tocopherols compounds in EE oil during deep-frying was found to be much lower than that in SE oil, which is highly consistent with their corresponding thermo-oxidative stabilities of the two oils during the fry. Thus, the study on the association between thermal-oxidation stability and antioxidant capacity may indicate that tocopherols in EE oil provide a stronger protection on oil stability during the deep-frying than the tocopherols in SE oil. The further analysis of phosphatidylcholines (PCs) content in the two oils found that the PCs content in EE oil is 19 times higher than that in SE oil. Considering that phosphatidylcholines function as an antioxidant synergist, it is proposed that the effect of synergistic characteristics between tocopherols and phospholipids plays the key roles for benefiting the thermo-stability of EE oil, but may occur less for the synergistic effect in SE oil due to its lower content of phosphatidylcholines. In summary, this study highlights the range of anti-oxidants in canola oil and the importance of appropriate extraction techniques in analyzing their protective capacity.

## Conflicts of interest

The authors declare no conflict of interest.

## Supplementary Material

RA-008-C8RA02275E-s001
