# Supplementary material for: Characterization of endogenous antioxidant attributes and its influence on thermal stability of canola oil
Source: RSC Adv. 2018 Oct 23;8(63):36096–103. doi: 10.1039/c8ra02275e (PMC9088397; doi:10.1039/c8ra02275e)
Supplement: RA-008-C8RA02275E-s001 [file RA-008-C8RA02275E-s001.pdf]

Electronic Supplementary Material (ESI) for RSC Advances.
This journal is © The Royal Society of Chemistry 2018

Fig. S1 Different content of individual phytosterols in two canola oils. Results were expressed as means ± standard deviations. Different lowercase letters above the bar indicate a significant difference ($P < 0.05$).

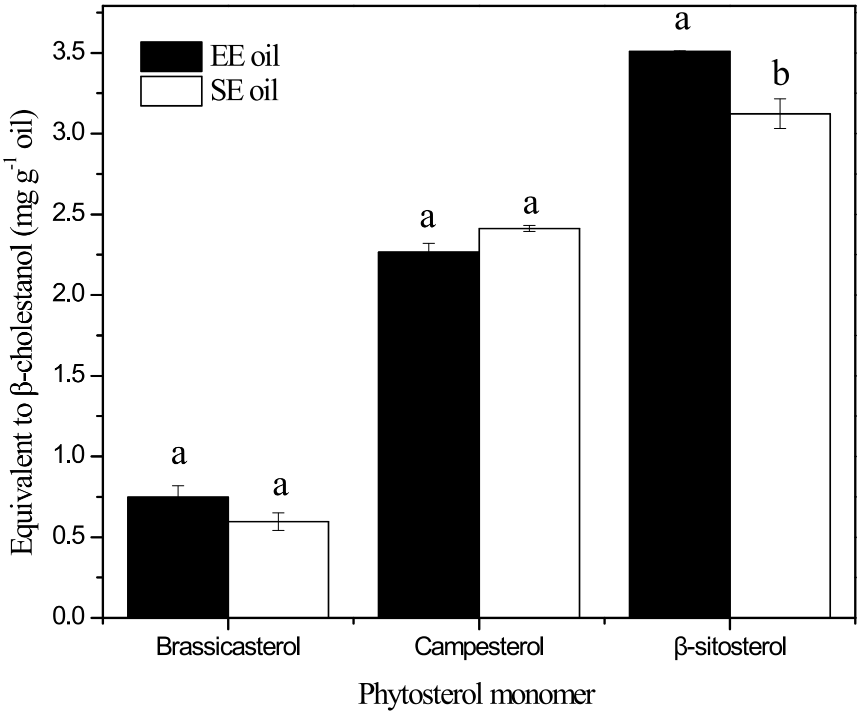

Table S1 Fatty acid composition of two canola oils at non-fried status (%).

Fatty acid	EE oil	SE oil
C14:0	0.08 ± 0.01[a]	0.05 ± 0.02[a]
C15:0	0.03 ± 0.01[a]	0.02 ± 0.01[a]
C16:0	4.01 ± 0.46[a]	3.90 ± 0.04[a]
C16:1	0.31 ± 0.04[a]	0.30 ± 0.01[a]
C17:0	0.09 ± 0.01[a]	0.08 ± 0.01[a]
C18:0	2.64 ± 0.30[a]	2.26 ± 0.22[a]
C18:1	59.47 ± 4.15[a]	69.39 ± 1.53[a]
C18:2	22.06 ± 0.32[a]	18.01 ± 0.62[b]
C20:0	0.91 ± 0.11[a]	0.61 ± 0.06[a]
C22:0	0.45 ± 0.05[a]	0.29 ± 0.01[b]
C24:0	0.16 ± 0.03[a]	0.14 ± 0.03[a]
∑SFA	8.36 ± 0.97[a]	7.35 ± 0.38[a]
∑MUFA	61.76 ± 3.87[a]	71.05 ± 1.62[a]
∑PUFA	22.41 ± 0.36[a]	18.30 ± 0.67[b]

Values are expressed as means ± standard deviations (n = 3). Different letters are significant in the same column at $P < 0.05$. SFA: saturated fatty acid, MUFA: monounturated fatty acids, PUFA: polyunturated fatty acids;
